# Selection and *In Vitro* Assessment of Plant Growth-Promoting Bacteria from Black Soldier Fly (*Hermetia illucens*) Frass

**DOI:** 10.1021/acsagscitech.5c00811

**Published:** 2026-02-12

**Authors:** Giovanni Lomonaco, Jeroen De Smet, Freek IJdema, Johan Ceusters, Francesco Iannielli, Rosanna Salvia, Mariana Amato, Carmen Scieuzo, Patrizia Falabella

**Affiliations:** † Department of Basic and Applied Sciences, 19006 University of Basilicata, Potenza 85100, Italy; ‡ Research Group for Insect Production and Processing, Department of Microbial and Molecular Systems, KU Leuven, Geel 2440, Belgium; § Department of Biosystems, Research Group for Sustainable Crop Production and Protection (SusCroPP), 26657 KU Leuven, Geel 2440, Belgium; ∥ Spinoff XFlies s.r.l., University of Basilicata, Potenza 85100, Italy; ⊥ Department of Agriculture, Forestry, Food and Environmental Sciences, University of Basilicata, Potenza 85100, Italy

**Keywords:** *Arabidopsis*, heat treatment, larval diet, phytohormone production, rhizobacteria, rhizosphere simulation, black soldier fly

## Abstract

Frass, the principal byproduct of Black Soldier Fly (BSF) farming, is increasingly valued as a sustainable organic fertilizer, partly due to its potential to harbor plant growth-promoting microorganisms (PGPM). This study investigated the presence and activity of PGPM in frass obtained from 10 rearing substrates and evaluated the effect of mandatory heat treatment (70 °C, 1 h). Using a rhizosphere-mimicking agar medium, 149 bacterial isolates were recovered and screened for PGPM-specific traits. Six promising isolates, belonging to *Serratia*, *Peribacillus*, *Acinetobacter*, *Pseudocitrobacter*, *Bacillus*, and *Enterobacter*, were further tested *in vivo* on *Arabidopsis thaliana*. They displayed variable effects on seed germination, root elongation, and root hair development linked to their phytohormone profiles. Several strains were recovered from both untreated and heat-treated frass, highlighting their thermotolerance. These findings demonstrate that BSF frass harbors PGPM with strong potential for biofertilizer development.

## Graphical abstract

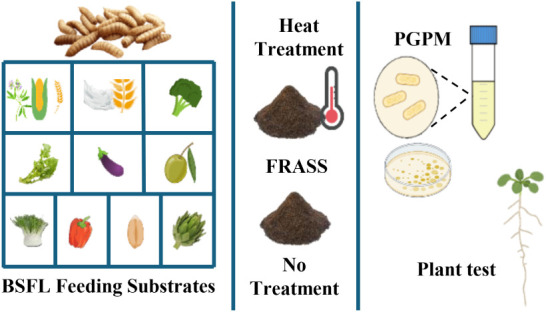

## 1 Introduction

Insect farming serves multiple purposes, including biological pest control, bait production, or the production of specific biomolecules (e.g., carmine red). However, many farms focus primarily on producing proteins and lipids for use in food and feed. In these cases, the bioconversion of organic substrates into biomass rich in proteins and lipids is of particular interest. Beyond their primary role, insect farms also produce valuable byproducts, such as frass—a nutrient-rich residue that can be repurposed as a soil amendment or fertilizer. Leveraging such byproducts not only reduces organic waste but also enhances the overall economic and environmental sustainability of the insect farming business model.^1–3^ Frass is defined by the EU Regulation 2021/1925 as “a mixture of excrements derived from farmed insects, the feeding substrate, parts of farmed insects, dead eggs, and with a content of dead farmed insects not exceeding 5% in volume and 3% in weight”. These leftovers are the primary byproduct of the bioconversion process^4^ and have been shown to have beneficial effects on plants.^5–7^ To exploit these benefits for plants, the EU Regulation 2021/1925 reports on the requirements for placing frass on the market as a fertilizer. Farmers are obliged to heat-treat the excrement at 70 °C for at least 60 min^8^ to ensure microbiological safety. Indeed, Van Looveren et al.^9^ demonstrated that heat treatment of frass can successfully lower the amount of unwanted microorganisms, guaranteeing compliance with the safety regulations specified by European Union standards. Although heat treatment guarantees the elimination of pathogens, it may also prevent microbial activity that could change its suitability as a soil fertilizer.^10^ Several studies have highlighted the presence of Plant Growth-Promoting Microorganisms (PGPM) in insect frass, framing it as a valuable resource for crop fertilization. However, these beneficial microbes may be compromised during the mandatory heat treatment.^11–14^ PGPM are a diverse group of microbes that inhabit the rhizosphere, the microenvironment surrounding the plant roots, and typically consist of arbuscular mycorrhizal fungi, rhizobia, and various plant growth-promoting (PGP) bacteria.^15^ They enhance plant development through multiple mechanisms, such as regulating phytohormone synthesis, improving soil nutrient availability, or increasing resistance to infections. Consequently, PGPM might reduce the need for artificial fertilizers, mitigate the impact of biotic and abiotic stressors, and boost plant yields.^16–18^ As a biofertilizer, PGPM can enhance nutrient availability by solubilizing soil minerals such as potassium and phosphorus, fixing atmospheric nitrogen, and producing phytohormones including auxins, cytokinins, and gibberellins.^12^ Plants can directly benefit from these phytohormones. For example, auxins regulate various physiological and developmental processes, such as root and shoot growth, cell expansion, vascular tissue differentiation, pathogen defense, and root colonization of microbes.^19–21^ Cytokinins play a crucial role in cell division, photosynthesis, chloroplast differentiation, control of leaf senescence,^22^ and nutrient metabolism.^23^ They also help maintain meristem activity, particularly in roots and shoots.^24,25^ Gibberellins primarily promote shoot growth but also influence several other developmental processes. They can accelerate leaves and fruits senescence, stop seed dormancy to stimulate germination, and enhance stem growth.^26,27^ At lower concentrations, gibberellins can also encourage root growth.^28^ Collectively, PGPM contribute to increased plant biomass, improved root development, greater plant height, enhanced seed germination and seedling vigor, higher chlorophyll content, increased photosynthetic rates, and expanded leaf area.^12^

Although several studies report beneficial effects of frass on various aspects of plant physiology, the outcomes clearly depend on factors such as frass composition, application rate, and treated plant species.^2^ At the same time, only a limited number of studies have investigated the actual presence of PGPM in frass or how their abundance influences the effectiveness of frass applications. Poveda et al.^11^ identified several genera of rhizobacteria within the microbial community of *Tenebrio molitor* frass, including *Pseudomonas*, *Acinetobacter*, *Pantoea*, and *Brevibacillus*—microbes known to promote plant growth through mechanisms such as auxin and gibberellin production, siderophore synthesis, and pathogen suppression. However, further research is needed, particularly for frass from the bioconverter *Hermetia illucens* (Black Soldier Fly, BSF). Previous studies have shown that the microbial community composition of the BSF frass varies with the larval diet,^29,30^ suggesting that the presence and abundance of PGPM may also differ, potentially leading to variable effects on plant growth. However, empirical data on PGPM occurrence and their PGP activities in BSF larval frass remain scarce. To address this knowledge gap between the presumed presence of PGPM and actual data, this study aimed to isolate PGPM from the frass of BSF larvae (BSFL),^31^ using a rhizosphere-mimicking agar. In addition, the influence of the feeding substrate, previously identified as a major driver of frass microbial composition,^29,32,33^ on the presence and abundance of PGPM was also explored. Frass derived from BSFL reared on 10 different diets was collected as the starting material for the isolation of microbes capable of colonizing the rhizosphere. Additionally, the effect of the heat treatment (1 h at 70 °C) on the presence and abundance of these microbes was assessed. In the second part of the study, the isolated bacteria were screened *in vitro* for several PGP traits: (I) growth in the presence of humic acids, (II) phosphorus solubilization, (III) ammonia production, and (IV) synthesis of phytohormones (auxins and gibberellins). The most promising isolates with PGP activities were identified using 16S rRNA gene sequencing, and the six most promising bacterial isolates were evaluated in an *in vivo* assay using *Arabidopsis thaliana* to assess their effect on germination and growth.

## 2 Material and Methods

### 2.1 BSFL Rearing

A schematic representation of the methods described in the following subsections is provided in the Supplementary Figure 1.

**Figure 1:**
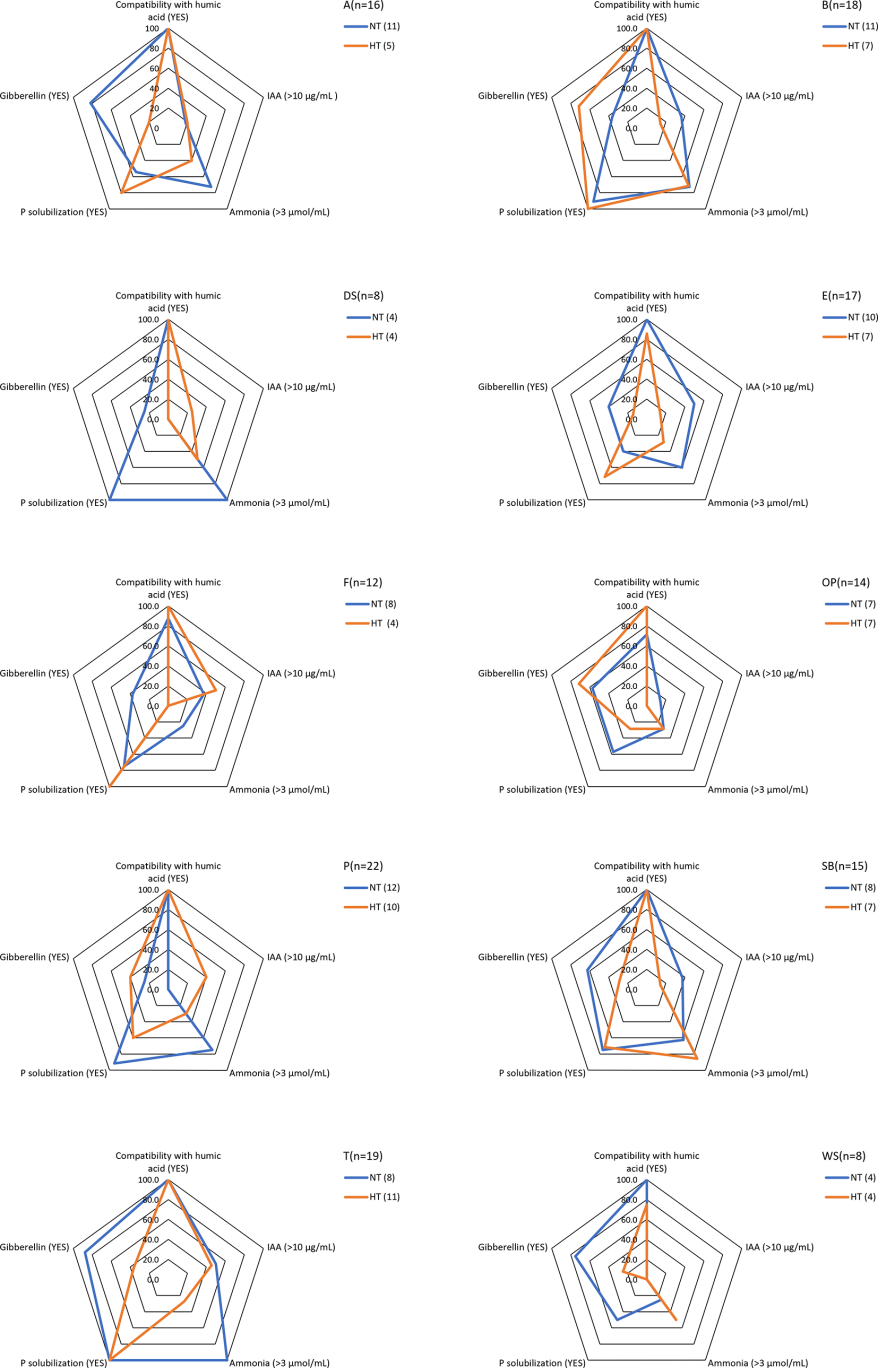
Characteristics of PGPM bacterial strains isolated from different frass types: peppers (P), broccoli (B), artichokes (A), fennel (F), turnip greens (T), eggplant (E), olive pomace (OP), seeds + whey (WS), spent barley (SB), and Gainesville diet (DS). For each frass type, isolates were obtained from both heat-treated (HT; treated at 70 °C) and nontreated (NT) samples. n indicates the total number of bacterial isolates screened per frass type, and percentages represent the proportion of isolates exhibiting a specific PGP trait within each group.

The frass used for the experiments was derived from the BSF colony reared by Xflies s.r.l. (Potenza, Italy). For 6 days, freshly hatched larvae were fed with a standard Gainesville diet (50% wheat bran, 20% maize meal, and 30% alfalfa).^34^ After 6 days, the 6-day-old larvae (6-DOL) were transferred to, and fed on, 10 different substrates consisting of vegetable byproducts: (i) peppers (P), (ii) broccoli (B), (iii) artichoke (A), (iv) fennel (F), (v) turnip greens (T), (vi) eggplant (E), (vii) olive pomace (OP), (viii) whey + seeds (WS), (ix) spent barley (SB) and (x) Gainesville diet (DS), according to the composition reported in Table 1. During the experiment, the BSFL were kept in complete darkness at a temperature of 27 ± 1 °C with 65 ± 2% relative humidity. Larvae were kept on these diets until 30% of the larvae reached the prepupal stage. At the end of the trial, larval frass from each diet was separated from the larvae by manual sieving using a 3 mm mesh. The collected frass was divided into two portions: one was HT for 1 h at 70 °C (HT) in accordance with EU Regulation 2021/1925, while the other remained NT. HT samples were subsequently stored at 16 °C until bacterial isolation and identification.

**Table 1:** Abbreviation and Composition of Diets Used in This Study

Abbreviation	Composition
**P**	35% peppers + 65% old bread
**B**	35% broccoli + 65% old bread
**A**	35% artichoke + 65% old bread
**F**	35% fennel + 65% old bread
**T**	35% turnip greens + 65% old bread
**E**	35% eggplant + 65% old bread
**OP**	35% olive pomace + 65% old bread
**WS**	43% sheep whey + 57% wheat seeds
**SB**	100% spent barley
**DS**	30% Gainesville diet + 70% water

### 2.2 Diet Preparation

The vegetable byproducts (peppers, broccoli, artichoke, fennel, turnip greens, and eggplant) were sourced from ARPOR (Scanzano Jonico, Matera, Italy), while olive pomace was obtained from F.lli PACE (Pietragalla, Potenza, Italy). Seeds and whey were provided by a farm in Muro Lucano (Potenza, Italy), and spent barley was sourced from BYKES BEER (Rivello, Potenza, Italy). The experimental diets consisted of a mixture of 35% vegetable substrate (peppers, broccoli, artichokes, fennel, turnip greens, eggplant, or olive pomace) and 65% surplus bread collected from the catering facilities of University of Basilicata, with a dry matter (DM) content of 22.0%. Bread was included to absorb excess moisture from vegetables, which could otherwise affect larval survival. Spent barley was used as received (DM = 22%), while the whey + seeds substrate consisted of 43% sheep whey and 57% wheat seeds in order to obtain a DM content of 22%.

The standard Gainesville diet (DM = 87.5%) was hydrated with water to achieve 70% moisture and served as the control.

### 2.3 Isolation of Bacteria from Frass

For the isolation of PGP bacteria in frass, Rhizosphere-Mimicking Agar (RMA), composed of synthetic root exudates, recalcitrant organic carbon sources, and salts, was prepared based on the method described by Brescia et al.^35^ with minor modifications (Supplementary Table 1). Specifically, all synthetic root exudates were sterilized using a 0.2 μm filter before being added to the RMA medium. Cycloheximide (100 mg/L) was added to the RMA medium.^36^ The pH of the RMA was adjusted to 6.5 prior to the addition of agar at a final concentration of 1.6% (w/v). A total of 3 g of frass was weighed and added to 27 mL of sterile saline solution (0.85% w/v NaCl) in sterile 50 mL tubes. The tubes were then shaken at 200 rpm for 1 h at room temperature. Following agitation, serial dilutions were prepared from 10^–1^ to 10^–7^. Aliquots of 100 μL from dilutions ranging from 10^–3^ to 10^–7^ were plated for microbial isolation. The prepared plates were incubated at 27 °C for 3 days. After 3 days, various colonies were randomly selected from different dilutions for all types of frass and both treatments. The selected bacteria were grown in Nutrient Broth at 27 °C for 1 day and subsequently stored in 50% glycerol at −20 °C and −80 °C.

### 2.4 Determining *In Vitro* Plant Growth-Promoting Activities

#### 2.4.1 Compatibility with Humic Acids

To assess compatibility with humic acids, the protocol reported by Vasseur-Coronado et al.^36^ was used with some modifications. The bacteria were grown in 5 mL of Nutrient Broth (Lab Lemco 1 g/L + Yeast extract 2 g/L + Peptone 5 g/L + NaCl 5 g/L) at 27 °C for 24 h with orbital shaking at 200 rpm. After 24 h, bacterial cultures were centrifuged at 4000 rpm for 2 min, and the pellets were resuspended in NaCl (0.85% w/v) to achieve a final optical density at 600 nm (OD600) of 0.1. Subsequently, the R2A (VWR Chemicals, Belgium) growth medium was prepared with the addition of 0.003% humic acid (Sigma-Aldrich, Switzerland). Once the plates were prepared (R2A + 0.003% humic acids), a volume of 5 μL was spotted at 3 points, and the test was repeated in triplicate. The plates were then incubated at 28 °C for 2 days.^36^

#### 2.4.2 Phosphate Solubilization

To assess the phosphate-solubilizing ability of the bacteria, Pikovskaya agar (Himedia, Germany) was used as the growth medium. For this analysis, the bacteria were grown in 5 mL of Nutrient Broth (Lab Lemco 1 g/L, Yeast extract 2 g/L, Peptone 5 g/L, NaCl 5 g/L) at 27 °C for 24 h with orbital shaking at 200 rpm. After 24 h, the bacterial cultures were centrifuged at 4000 rpm for 2 min, and the resulting pellets were resuspended in 0.85% (w/v) NaCl to achieve a final optical density at OD600 of 0.1. Once the plates were prepared, 5 μL of the bacterial suspension was spotted in three separate locations, and the test was performed in triplicate. The plates were then incubated at 28 °C for 2 days.^36^ The development of a halo around the bacterial colony indicates the bacterium’s ability to solubilize phosphate.

#### 2.4.3 Ammonia Production

For ammonia production, bacteria were cultured in sterile 15 mL tubes containing 5 mL of Nutrient Broth (Lab Lemco 1 g/L, Yeast extract 2 g/L, Peptone 5 g/L, NaCl 5 g/L), incubated at 27 °C on an orbital shaker at 200 rpm. Overnight cultures (50 μL, OD600 = 0.2) were inoculated into 5 mL of 1% peptone broth (HiMedia) and incubated at 37 °C in a shaking incubator at 150 rpm for 48 h. The cultures were then centrifuged at 3000 rpm for 5 min. For quantitative ammonia estimation, 1 mL of Nessler’s reagent (Chemlab, Belgium) was added to 0.2 mL of cell-free supernatant, followed by 8.5 mL of distilled water. The spectrophotometric reading with a Thermo Scientific Genesys 10S UV–vis (Thermo Fisher Scientific, Waltham, MA, USA) was immediately taken at 450 nm. Uninoculated 1% peptone broth mixed with 8.5 mL of distilled water and 1 mL Nessler’s reagent was used as a blank.^37^ The ammonia concentration was determined using a standard curve of ammonium sulfate with concentrations in the range of 0.6–8 μmol/mL.

#### 2.4.4 Indole-3-Acetic Acid Production

For indole-3-acetic acid (IAA) production, bacteria were grown in sterile 15 mL tubes containing 5 mL of Nutrient Broth (Lab Lemco 1 g/L, Yeast extract 2 g/L, Peptone 5 g/L, NaCl 5 g/L) and incubated at 27 °C on a shaker set to 200 rpm. A 50 μL overnight culture with an OD600 of 0.2 was transferred into 5 mL of LB broth (prepared by mixing 10 g of tryptone, 5 g of yeast extract, and 10 g of NaCl in 1 L of distilled water, adjusting the pH to 7.0 using 1 N NaOH, and sterilizing at 120 °C for 25 min), supplemented with 0.1% tryptophan. The cultures were incubated for 3 days at 30 °C.^38^ Following incubation, the cultures were centrifuged at 3000 rpm for 5 min, and 2 mL of the supernatant was collected. This was combined with 4 mL of Salkowski’s reagent (50 mL of 35% perchloric acid and 1 mL of 0.5 M FeCl_3_ solution)^39^ and incubated for 10 min.^38,40^ A blank was prepared by mixing 2 mL of uninoculated LB broth containing tryptophan with 4 mL of Salkowski’s reagent. The color intensity was measured using a spectrophotometer at 535 nm (Thermo Fisher Scientific, Waltham, MA, USA), and the IAA production for each sample was calculated using a standard IAA curve with concentrations ranging from 1 to 100 μg/mL.

#### 2.4.5 Gibberellin Production

For gibberellin production, bacteria were grown in sterile 15 mL tubes containing 5 mL of Nutrient Broth (Lab Lemco 1 g/L, Yeast extract 2 g/L, Peptone 5 g/L, NaCl 5 g/L) and incubated at 27 °C on a shaker set to 200 rpm. A 50 μL overnight culture with an OD600 of 0.2 was transferred into 20 mL of Nutrient Broth (NB, Oxoid) at 27 °C on an orbital shaker (200 rpm) for 5 days. After the incubation period, the tubes were centrifuged at 4000 rpm for 5 min, and 15 mL of supernatant was taken and placed in a new tube. In addition to 15 mL of supernatant, we added 2 mL of zinc acetate reagent (21.9 g of zinc acetate +1 mL of glacial acetic acid, and the volume was made up to 100 mL with distilled water), and after 2 min, we added 2 mL of potassium ferrocyanide (10.6% in distilled water). After that, it was centrifuged at low speed (2000 rpm) for 15 min. Five mL of supernatant and 5 mL of 30% HCI were added, and the mixture was incubated at 20 °C for 75 min.^41^ For the blank, 5 mL of 30% HCl was used. Absorbance was read at 254 nm (Thermo Fisher Scientific, Waltham, MA, USA). The concentration of gibberellins was calculated by preparing a standard curve by using gibberellic acid (GA3, Hi-Media) as the standard (at concentrations of 100–1000 μg/mL).

### 2.5 Isolate Identification Using 16S rRNA Gene Sequencing

Bacterial strains exhibiting beneficial properties were identified. Selected isolates were grown overnight on Plate Count Agar (PCA) from the stock culture collection. From each plate, one individual colony was suspended in 20 μL of Milli-Q water, and the genomic DNA from each isolate was released through cell lysis by boiling. Next, the bacterial 16S rRNA region was amplified using the primers 27F (5′-AGA GTT TGA TCM TGG CTC AG-3′) and 1492R (5′-CTA CGG CTA CCT TGT TAC GA-3′), as previously described by Gorrens et al.^53^ PCR was carried out with a DreamTaq DNA polymerase (Thermo Scientific) according to the manufacturer’s protocol. The PCR program was as follows: initial denaturation of 3 min at 95 °C, followed by 30 cycles of 30 s at 95 °C, 30 s at 50 °C, 2 min at 72 °C, and a final extension step of 10 min at 72 °C. A negative control, for which the DNA was replaced with sterile Milli-Q water, was included in each PCR run. Specificity of the 16S rRNA gene amplification was checked using gel electrophoresis, and if a single band was observed, the PCR product was purified using the GeneJet PCR purification kit. The obtained PCR products were sent for Sanger sequencing at Eurofins Genomics, after which the obtained sequences were identified by using BLASTn.

### 2.6 Inoculation of *Arabidopsis* Seeds with Promising Bacteria

From the identified isolates, the six bacteria with the best *in vitro* PGP characteristics were selected to analyze their effects on the plant species *Arabidopsis thaliana*. For the *in vivo* assay on *Arabidopsis thaliana*, bacteria were grown in LB medium at 28 °C for 24 h with shaking at 200 rpm. The seeds were surface sterilized using 70% ethanol for 2 min, followed by 5% hypochlorite for 2 min, and then washed five times with sterile bidistilled water. Subsequently, the seeds were immersed in the bacterial solution (OD 0.3) for 1.5 h under orbital agitation.^43^ The seeds were then collected and placed on agar medium composed of a standard solution^44^ with the addition of 1% agar. To assess germination and root elongation, the plates were placed vertically in a growth chamber. The growth conditions were as follows: 23 °C/18 °C light/dark temperature; light/dark photoperiod of 16/8 h; photosynthetically active radiation of 130 μmol m^–2^ s^–1^, and a relative humidity (RH) of 70%. Germination was monitored every 12 h from the beginning of the assay by counting the number of germinated seeds for each treatment. After 5 days, the seedlings were collected to measure root length, stem length, and the number of root hairs per mm of root length with ImageJ 1.54d software.

### 2.7 Identification of the Bacterial Community in the Frass Samples Using 16S rRNA Gene Sequencing

From each of the 10 BSF frass types, three replicate samples of approximately 250 mg were taken and subjected to DNA extraction using the E.Z.N.A. Soil DNA Kit (Omega Bio-Tek, Norcross, GA, USA), following the manufacturer’s instructions. The concentration and purity of the DNA were verified using a NanoDrop device (ND-1000, Isogen Life Science, Utrecht, The Netherlands) before the samples were sent to Novogene Bioinformatics Technology Co., Ltd. for sequencing of the V4 region of the 16S rRNA gene using barcoded primers 515F (5′-GTG CCA GCM GCC GCG GTA A-3′) and 806R (5′-GGA CTA CHV GGG TWT CTA AT-3′). The PCR procedure and bioinformatic analysis of the raw sequencing data are described in detail in Van Looveren et al.^42^ In short, paired-end 16S rRNA gene V4 region amplicon sequencing was merged and filtered before clustering. The UNOISE algorithm was used to cluster sequences into zero-radius operational taxonomic units (zOTUs), followed by taxonomic classification using the SINTAX algorithm with a bootstrap confidence value of 0.80. The reads were further filtered by removing those with less than 0.1% abundance in each sample. Differences in bacterial communities among frass types were evaluated using the alpha diversity indices Chao1 and Shannon’s diversity index. Beta diversity estimates based on Hellinger-transformed Bray–Curtis distances were analyzed using a permutational analysis of variance (PERMANOVA) and visualized in a Nonmetric Multidimensional Scaling (NMDS) plot, using the phyloseq and MicroViz packages in R version 4.3.0. The mean relative abundance of the bacterial community was further visualized in a dot plot using the ggplot2 package in R.

### 2.8 Statistical Analysis

For the six best bacteria, the measurement was carried out three times, while for the plant growth parameters, measurements were carried out 10 times. The normality of the data was assessed by using the Shapiro–Wilk test. Data were analyzed by one-way ANOVA and Tukey’s *post hoc* test at p = 0.05. Statistical analyses were performed using a GraphPad Prism version 6.0.0 for Windows (GraphPad Software, San Diego, CA, USA).

## 3 Results

### 3.1 Determining the Load of Microbes Capable of Growing in the Rhizosphere

Plating the different types of frass on a RMA revealed a high number of potential bacteria (between 4.57 log colony-forming unit (CFU)/g frass and 8.08 log CFU/g frass) from frass in which this number clearly varied depending on the frass type, with NT OP frass outperforming all other types (Supplementary Figure 2). Interestingly, the effect of a heat treatment on this number was rather limited, with 6.23 log CFU/g frass still persisting in the HT samples. Specifically, the rhizobacterial population decreased only slightly from 6.52 log CFU/g in the NT frass to 6.23 log CFU/g after treatment, corresponding to a retention of approximately 95.6% of the rhizobacteria. These values represent the average across all tested frass types. This suggests that the applied heat treatment had a minimal impact on the overall survival of the rhizobacteria.

### 3.2 Isolation and Assessment of Potential Plant Growth-Promoting Bacterial Strains

The total CFU number was quantified for each frass type. Using the same RMA agar, 149 bacterial isolates were randomly selected from the larger pool of colonies that developed (Supplementary Figure 3), originating from the 10 different types of frass (either HT or NT) for further testing. These 149 isolates were subjected to *in vitro* screening of PGP activities (Figure 1). The assessed parameters for the different bacterial isolates were as follows: (I) compatibility with humic acids, (II) phosphate solubilization ability, (III) ammonia production, and (IV) the synthesis of IAA and gibberellins. For certain parameters, such as compatibility with humic acids, phosphate solubilization ability, and gibberellin production, the assessment was qualitative, determining whether the bacteria had this ability or not. In contrast, ammonia and IAA production were quantified. Ammonia production was then scored as either greater or less than 3 μmol/mL, while IAA production was evaluated as either greater or less than 10 μg/mL.

An overview of the results obtained for all tested bacteria for the different types of frass can be found in Figure 1. For each type of frass, the same graph also compares the results for bacteria isolated from HT (orange) and NT (blue) frass. Important differences were observed between HT and NT of the same frass typology. In all frass types, humic acid compatibility consistently reached 100% for both HTand NT isolates. This might indicate that this parameter was not affected by heat treatment but is more likely the result of an initial selection for isolates with a tolerance to humic acids due to their presence in the RMA. Phosphorus solubilization showed variability between different frass types and treatments. A, B, P, WS, DS, OP, and SB frass showed consistently more isolates in NT frass than in HT frass, which suggests that heat treatment reduced the number of phosphorus-solubilizing bacteria. In E and F frass, more isolates capable of phosphorus solubilization were present in the HT frass than in NT frass, while in T frass, all selected bacteria were able to solubilize phosphate. The presence of isolates able to produce ammonia also differed between different frass types. For example, in samples A, B, DS, E, F, P, and T, NT frass showed a higher prevalence of isolates with higher ammonia production than HT frass, whereas in samples WS and SB, the opposite was observed. Additionally, a general trend toward reduced IAA production in the isolates was observed in the HT frass samples. This was particularly evident in samples A, B, E, OP, and SB, where bacteria isolated from NT exhibited higher IAA production compared to those isolated from HT frass. However, in certain cases, such as DS, P, F, and T, the values were relatively similar, suggesting that some IAA-producing bacterial strains might be more resistant to heat treatment. Furthermore, all bacteria isolated from both T- and NT-WS strains produced IAA at levels below 10 μg/mL. A notable enrichment in IAA-producing strains was observed in frass E and SB, particularly in NT conditions. There was also a large variability in the ability to produce gibberellins. In some frass types, such as A, T, DS, E, F, WS, and SB, heat treatment appeared to reduce the number of bacteria capable of producing gibberellins. In other frass samples, such as B, OP, and P, most of the bacteria selected after HT were able to produce gibberellins when compared with bacteria selected from NT frass. Ammonia production was relatively higher in bacteria isolated in frass B, OP, and T, although it decreased in HT samples. One of the important observations is that heat treatment tended to reduce the occurrence of most PGP traits, including IAA and gibberellin production, phosphate solubilization, and ammonia release, suggesting a detrimental impact on beneficial microbial functions. However, no full reduction was observed either, indicating a significant fraction of PGPMs are able to survive controlled heat treatments.

### 3.3 Sequencing Results of the Best Performing Bacteria Isolated from Treated and Untreated Frass

Out of the 149 bacterial isolates, 73 were identified using full 16S rRNA gene sequencing. Because the colonies were randomly selected for this preliminary functional screening, these identified isolates represent only the subset showing relevant PGP activities and do not provide a comprehensive or quantitative characterization of the frass microbiota. Their selection was based on their overall performance across all evaluated PGP parameters, including indole-3-acetic acid (IAA) production, ammonia production, phosphate solubilization, and gibberellin production. Strains showing the highest activity in one or more of these assays were retained for molecular identification, and results are depicted for the isolates from the HT (Figure 2) and NT (Figure 3) frass separately. In both figures, the bacterial family, genus, and type of frass from which they were isolated are also depicted. For the NT frass (Figure 2), the most abundant bacterial family is Moraxellaceae, of which *Acinetobacter* is the sole genus identified. This genus was found across several types of frass, including F, T, E, SB, A, and P. The second most represented bacterial family is Enterobacteriaceae, which includes four distinct genera: *Enterobacter*, *Cronobacter*, *Pseudocitrobacter*, and *Klebsiella*. Interestingly, bacteria belonging to the genera *Enterobacter*, *Cronobacter*, and *Pseudocitrobacter* have been selected from the B frass. The third most representative family is Yersiniaceae, represented by the genus *Serratia*. Other families of bacteria have been isolated, but their presence varies depending on the type of frass.

**Figure 2:**
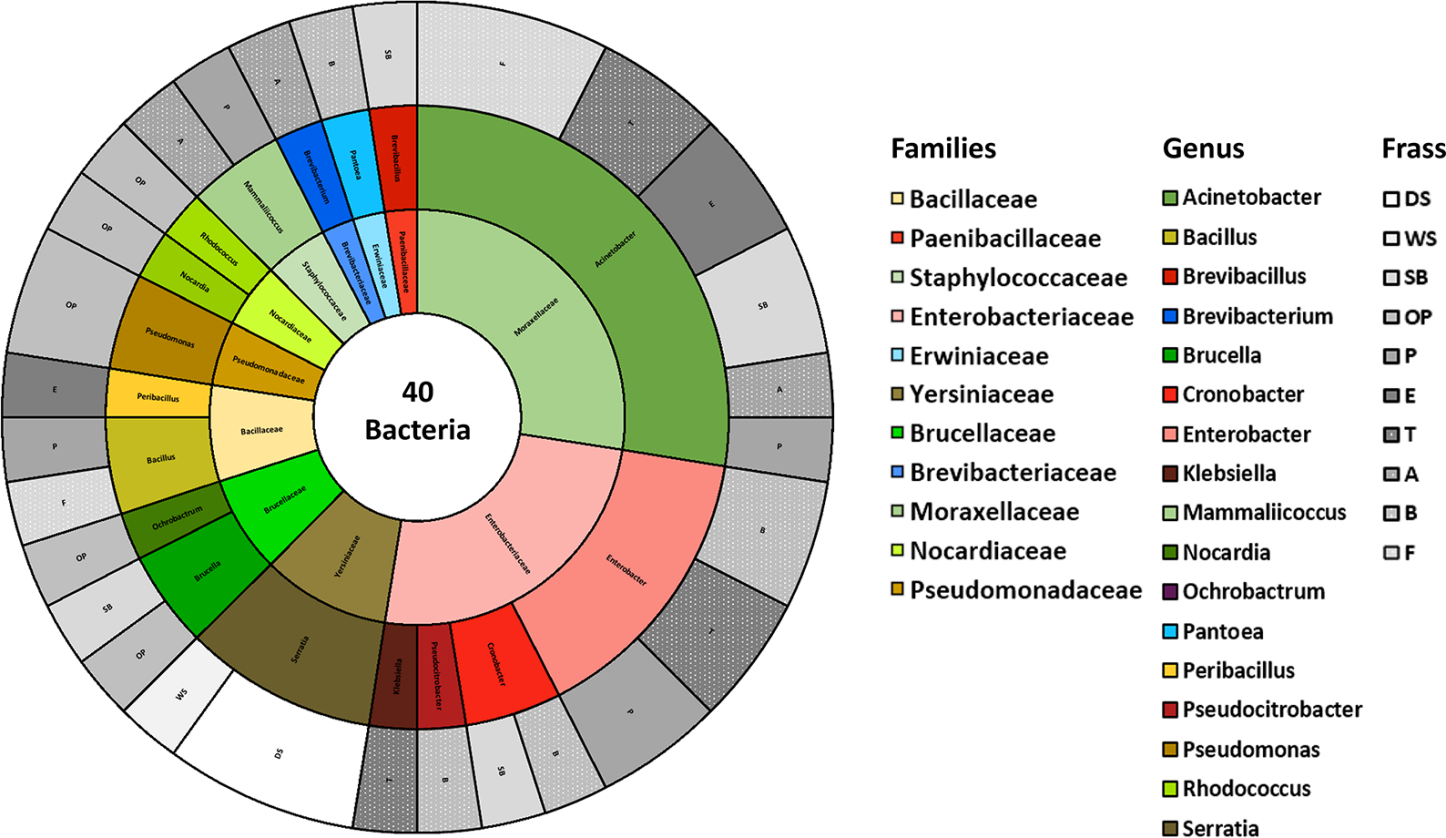
Overview of the identification for the 40 bacterial isolates retrieved from NT frass at the family level (inner circle) and genus level (middle circle); the outer circle depicts the type of frass they have been isolated from.

**Figure 3:**
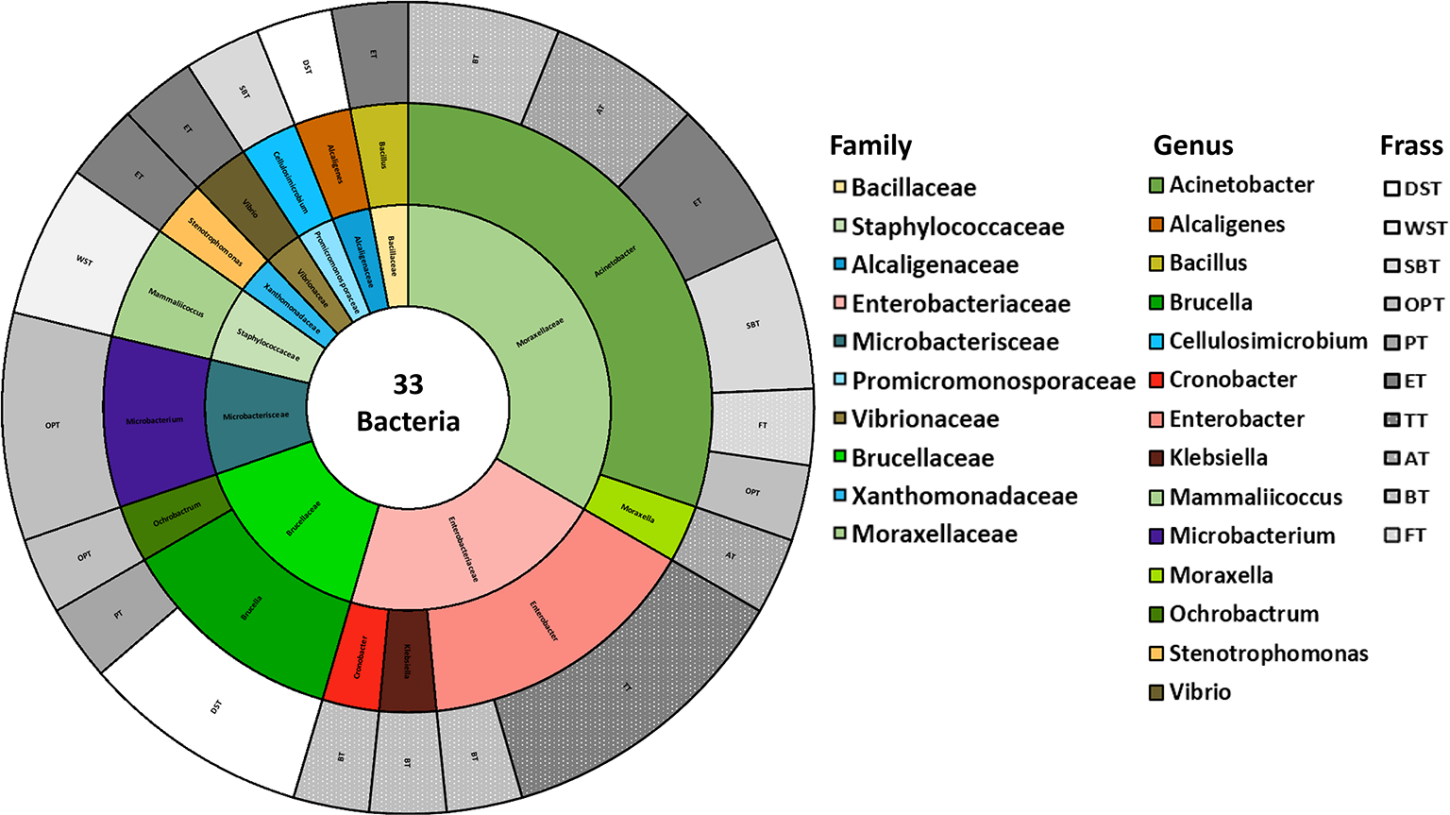
Families (inner circle) and genera (middle circle) of the 33 bacterial isolates identified and isolated from different types of HT frass (outer circle).

Looking at the HT frass samples (Figure 3), the two most identified families remain the Moraxellaceae and Enterobacteriaceae. While the Yersiniaceae do not seem to survive the heat treatment, the Brucellaceae do and become the third most abundant family. For the family Moraxellaceae, in addition to the genus *Acinetobacter*, the genus *Moraxella* was also identified in bacteria isolated from AT frass. In the family Enterobacteriaceae, there are no bacteria belonging to the genus *Pseudocitrobacter*, compared to the results of the NT frass. The genera *Brucella* and *Ochrobactrum* belong to the family Brucellaceae, and these were isolated from several frass types such as DST, PT, and OPT.

As observed from the comparison between Figures 2 and 3, the genera most commonly found in NT frass were also present in HT frass, including *Bacillus*, *Acinetobacter*, *Mammalicoccus*, *Cronobacter*, *Enterobacter*, *Klebsiella*, and *Brucella*. This indicates that the HT applied to the frass did not have significant effects on these bacterial groups. Some families were present in the NT frass but not in the HT samples, such as Paenibacillaceae, Yersiniaceae, Brevibacteriaceae, Nocardiaceae, and Pseudobacteriaceae. Conversely, in the HT frass, certain bacterial families were identified even if they were absent in the NT samples, including Alcaligenaceae, Microbacteriaceae, Promicromonosporaceae, and Xanthomonadaceae.

### 3.4 Characteristics of the Six Selected Bacterial Isolates with the Most Potent PGP Activity

The six bacteria selected from among those having the most potent rhizobacterial characteristics, as well as their ability to grow in the presence of humic acids, solubilize phosphate, and produce gibberellins, are depicted in Table 2.

**Table 2:** Six Bacterial Strains Selected for High Performance in Preliminary Screening for PGP Traits^a^

Bacteria	Compatibility with humic acids	Phosphate solubilization	Gibberellin production
*Serratia* sp.	+	+	–
*Peribacillus* sp.	+	+	–
*Acinetobacter* sp.	+	+	+
*Pseudocitrobacter* sp.	+	–	+
*Bacillus* sp.	+	+	+
*Enterobacter* sp.	+	+	–
*Paenibacillus polymyxa*	+	–	–

a Compatibility with humic acids: (−) Not able to grow in the presence of humic acids; (+) Able to grow in the presence of humic acids. Phosphate solubilization: (−) Absence of solubilization halo; (+) Presence of solubilization halo. Gibberellin production: (−) Not able to produce gibberellins; (+) Able to produce gibberellins. Data are results of three independent PGP assays shown in separate columns, each performed in triplicate.

As a reference for comparison, the rhizobacterium *Paenibacillus polymyxa* was included, as it is documented in the literature for its excellent rhizobacterial properties.^45^ The selected bacteria *Acinetobacter* sp. and *Bacillus* sp. had the broadest activity within the evaluated parameters. *Serratia* sp., *Peribacillus* sp., and *Enterobacter* sp. are unable to produce gibberellins but demonstrate high compatibility with humic acids and the ability to solubilize phosphate. Conversely, *Pseudocitrobacter* sp. lacks phosphate-solubilizing capacity but is capable of producing gibberellins and growing in the presence of humic acids. The control strain *P. polymyxa* did not exhibit a phosphate-solubilizing ability, which differs from what has been previously reported in the literature.

Next, the initial screening for ammonia and auxin production was assayed to evaluate variability among these six isolates (Figure 4). In absolute terms, *Bacillus* sp. exhibited the highest ammonia production (Figure 4a), showing a significant difference compared to *P. polymyxa* and *Serratia* sp. However, no significant differences were observed among the other selected bacterial strains. The *Bacillus* sp. also exhibited the highest IAA production, together with the *Enterobacter sp*. (Figure 4b), showing a significant difference compared to those of *Peribacillus* sp. and *Acinetobacter* sp.

**Figure 4:**
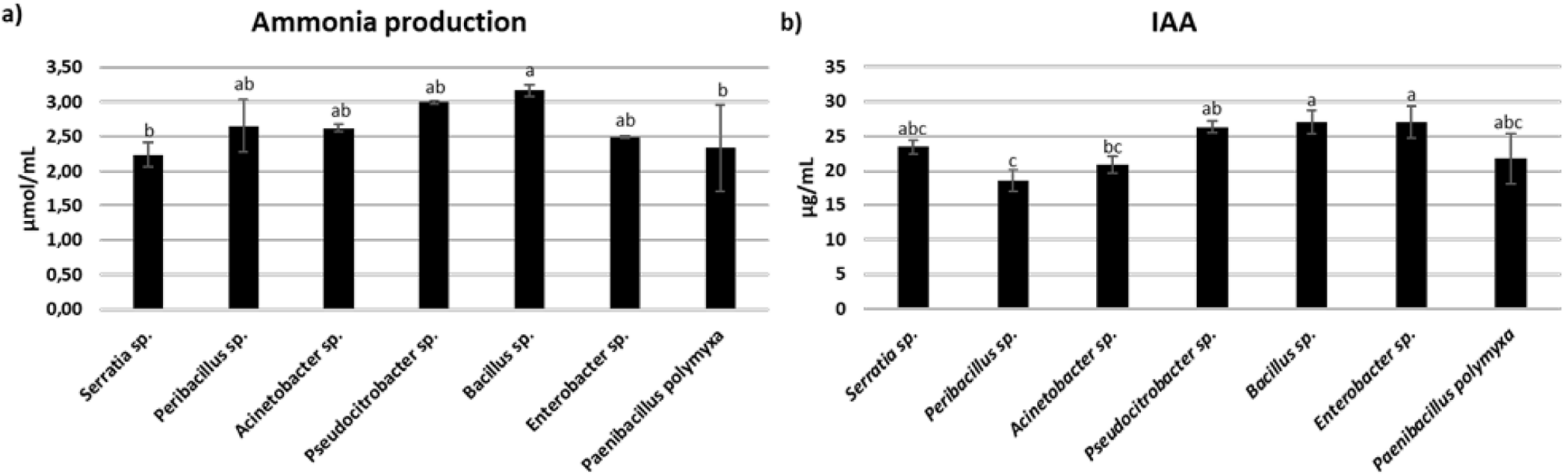
Ammonia (a) and indole-3-acetic acid (IAA) production for the six selected bacterial strains (b). Different letters designate significantly different values. Data are presented as the mean of replicates (*N* = 3) ± SD (Standard Deviation), significantly different according to one-way ANOVA followed by Tukey’s *post hoc* test (*p* < 0.05).

### 3.5 Effects on Germination and Early Plant Growth of *Arabidopsis thaliana*

To evaluate whether these *in vitro* tests accurately predict an effect on plant growth, the impact of the selected isolates on the germination process of *Arabidopsis thaliana* seeds was explored (Supplementary Figure 4), with germination assessments conducted every 12 h after seed placement on the plates. Differences in seed germination were evident after 36 and 48 h. After that period, no differences between treatments were observed anymore. Seeds treated with *P. polymyxa* exhibited the highest germination percentage after 36 h compared to the other treatments (Figure 5). However, after 48 h, the differences were no longer statistically significant, with *P. polymyxa* only showing a significant difference compared to *Bacillus* sp. and *Acinetobacter* sp.

**Figure 5:**
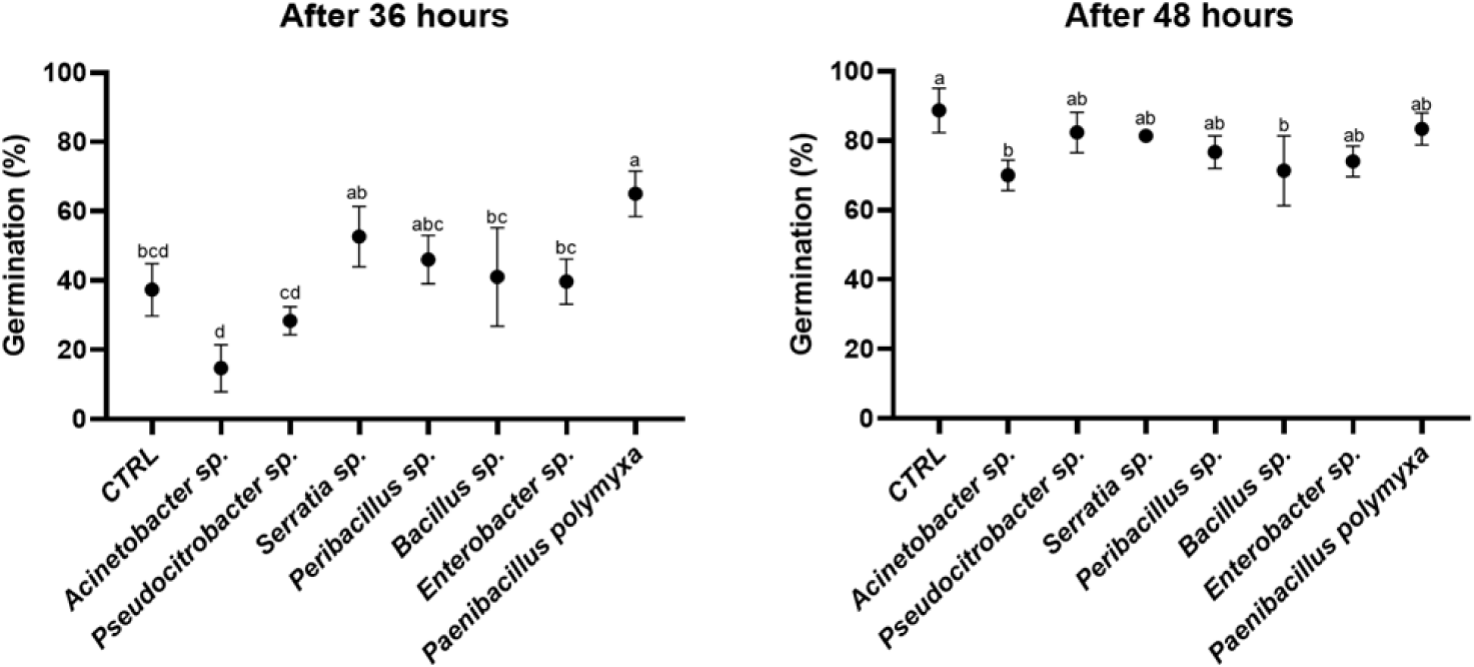
Effect of germination of seeds inoculated with selected strains after 36 and 48 h. Different letters designate significantly different values. Data are presented as the mean of replicates (*N* = 3) ± SD (Standard Deviation), significantly different according to one-way ANOVA followed by Tukey’s *post hoc* test (*p* < 0.05).

In addition to germination, three further parameters were evaluated after 5 days: stem length, root length, and the number of root hairs per millimeter of root length (Figures 6a, b, and 7, respectively). Notably, for the stem length (Figure 6a), the control treatment (bacterial growth medium) showed only a significant difference compared to the *P. polymyxa* treatment, while no significant differences were observed in comparison to all other treatments. For the root length, on the other hand, a significant reduction in root elongation was observed in plants treated with four bacterial isolates: *Acinetobacter* sp., *Pseudocitrobacter* sp., *Enterobacter* sp., and *P. polymyxa*, when compared to the untreated control. This suggests that these strains may exert inhibitory effects on early root development under the tested conditions. In contrast, no statistically significant differences in root length were found between the control and the other bacterial treatments, indicating that the remaining isolates did not negatively impact root elongation. These results highlight the variable influence of different bacterial strains on root growth dynamics and emphasize the importance of strain-specific screening when evaluating plant–microbe interactions.

**Figure 6:**
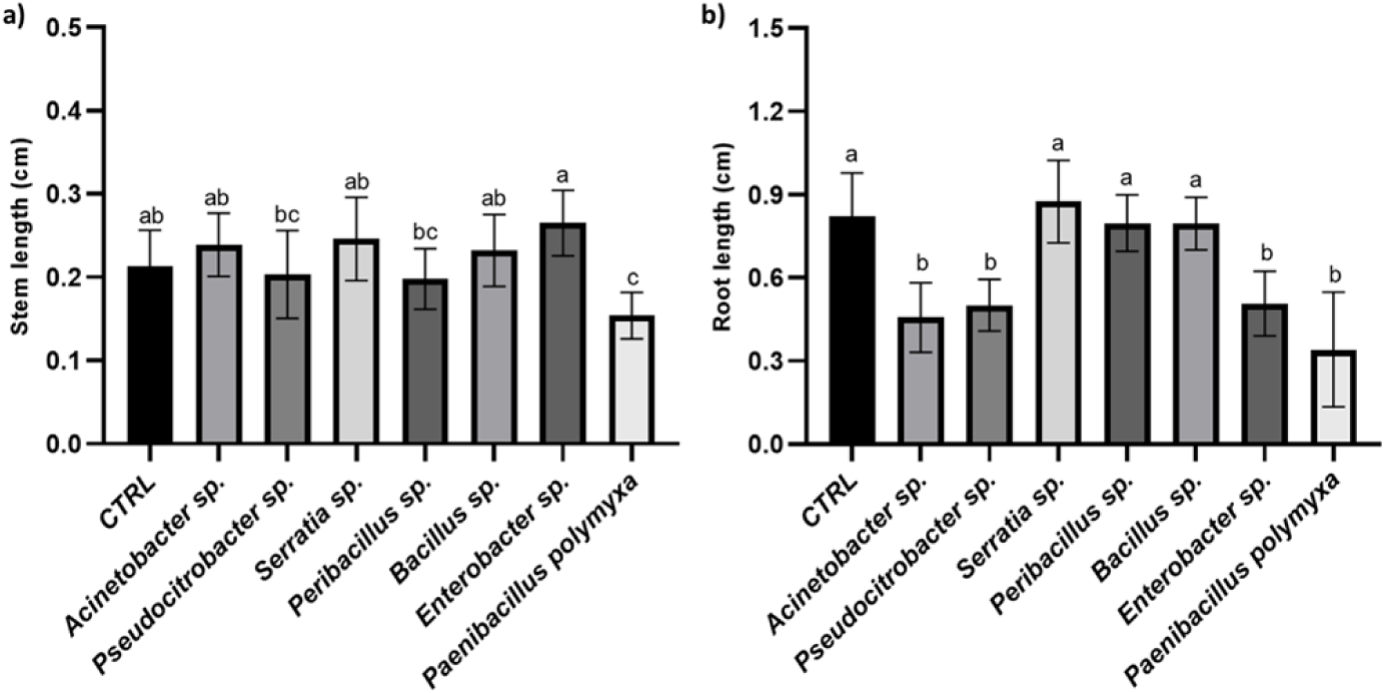
Stem length (a) and root length (b) measured after 5 days. Different letters designate significantly different values. Data are presented as the mean of replicates (*N* = 10) ± SD (Standard Deviation), significantly different according to one-way ANOVA followed by Tukey’s *post hoc* test (*p* < 0.05).

**Figure 7:**
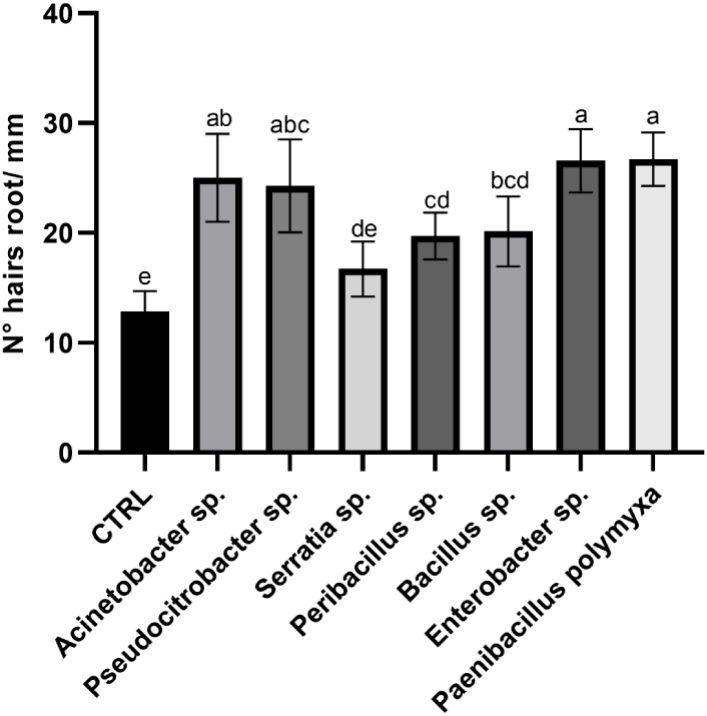
Number of root hairs/mm. Different letters designate significantly different values. Data are presented as the mean of replicates (*N* = 10) ± SD (Standard Deviation), significantly different according to one-way ANOVA followed by Tukey’s *post hoc* test (*p* < 0.05).

Finally, interesting differences were observed in the number of root hairs per millimeter of root length (Figure 7). To illustrate this, a microscope image of the control condition versus the *Acinetobacter* sp. inoculated treatment is depicted in Figure 8. Treatments that resulted in reduced root length tended to have a higher number of root hairs compared to the control treatment. This was the case for *Acinetobacter* sp. and *Pseudocitrobacter* sp., which, despite not promoting root length, induced a notable increase in root hair formation. Similarly, *Enterobacter* sp. and *P. polymyxa* also showed high root hair density. These findings suggest that isolates promoting root hair development, regardless of their effect on elongation, may be promising candidates for enhancing nutrient and water uptake in plants and, thus, hold agronomic potential in the long term. On the other hand, two bacteria that did not reduce root length had an increased number of root hairs compared to the control, being *Peribacillus* sp. and *Bacillus* sp. Indeed, the control exhibited a significantly lower number of root hairs compared to all treatments except for *Serratia* sp. The treatments that showed the highest number of root hairs in absolute value were *P. polymyxa* and *Enterobacter* sp.

**Figure 8:**
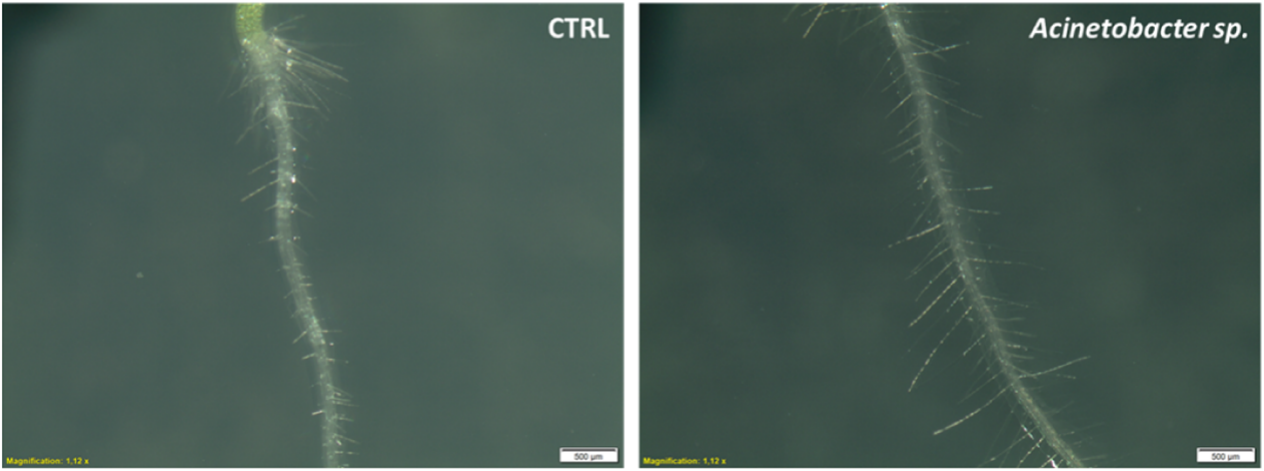
Photomicrograph of roots of control (left) and *Acinetobacter sp* (right) with root hairs.

### 3.6 Determining the Presence and Abundance of the Six Most Promising Isolates in Different Frass Types

To get a sense of how abundant and widespread these promising isolates are across the frass originating from the different feeds, the complete bacterial community of the 10 frass types was analyzed through sequencing of the V4 region of the 16S rRNA gene. PERMANOVA analysis revealed significant differences in bacterial community composition among frass types (*R* ^2^ = 0.980, *p* = 0.001), although some frass types exhibited greater similarity than others (Figure 9). For example, frass types E and F exhibited relatively low species richness and evenness (Figure 10) and were dominated by zOTU3 (28.9% and 23.1%) and zOTU5 (13.22% and 10.0%), respectively (Figure 11, Table 3). In contrast, frass types DS and SB displayed high species diversity and were characterized by the presence of zOTU18 (7.8% and 3.4%) and zOTU19 (8.4% and 2.6%), which were absent in the other frass types. Finally, frass types B and T exhibited similar bacterial composition, with zOTU1 comprising the largest proportion of the microbiome (26.6% and 24.8%), followed by zOTU2 (16.3% and 15.0%, respectively).

**Figure 9:**
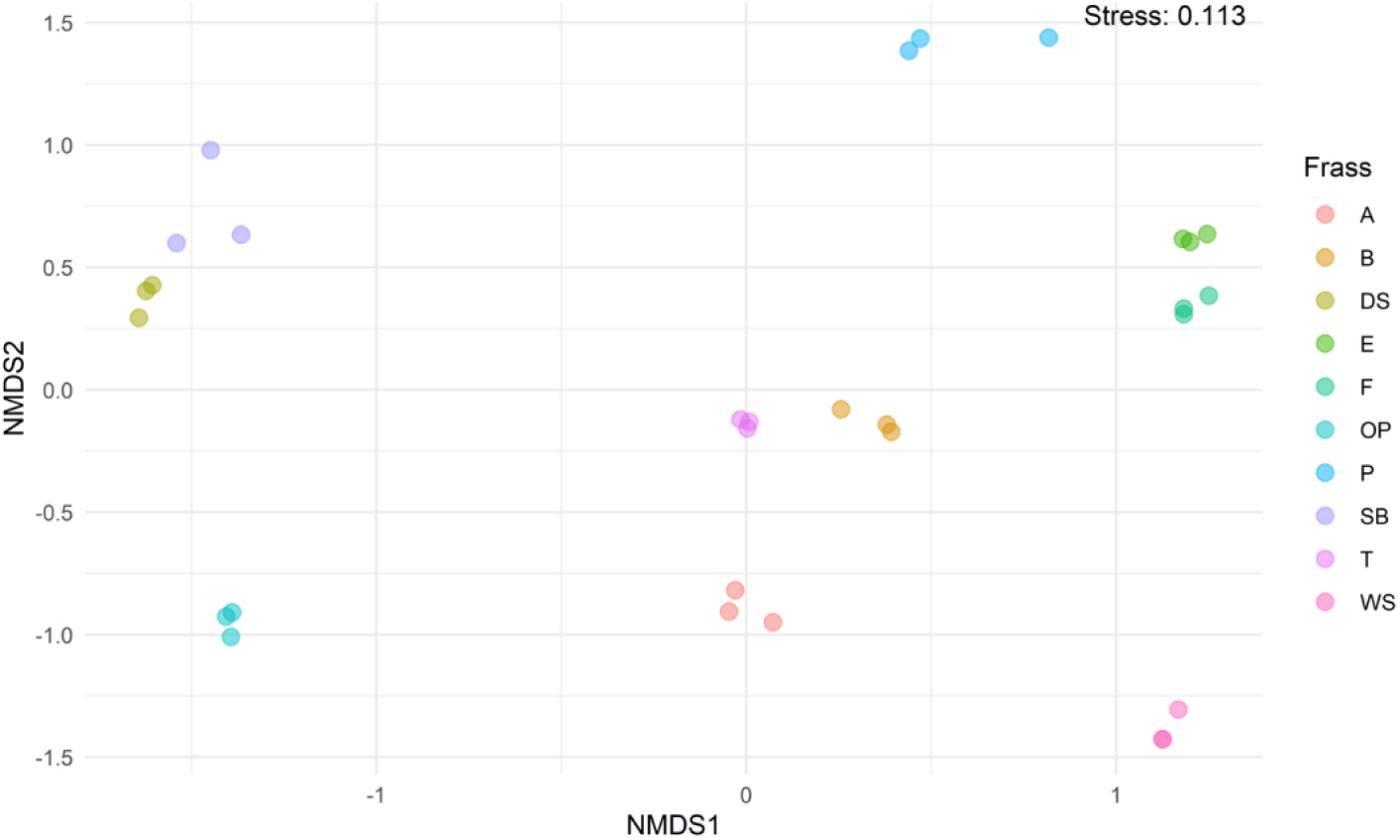
Ordination plot of 10 different frass types. Nonmetric multidimensional scaling (NMDS) of 30 frass samples is based on the Bray–Curtis dissimilarities of the Hellinger-transformed relative abundance data. Colors indicate the different frass types, and the stress value of the NMDS ordination is shown in the upper-right corner.

**Figure 10:**
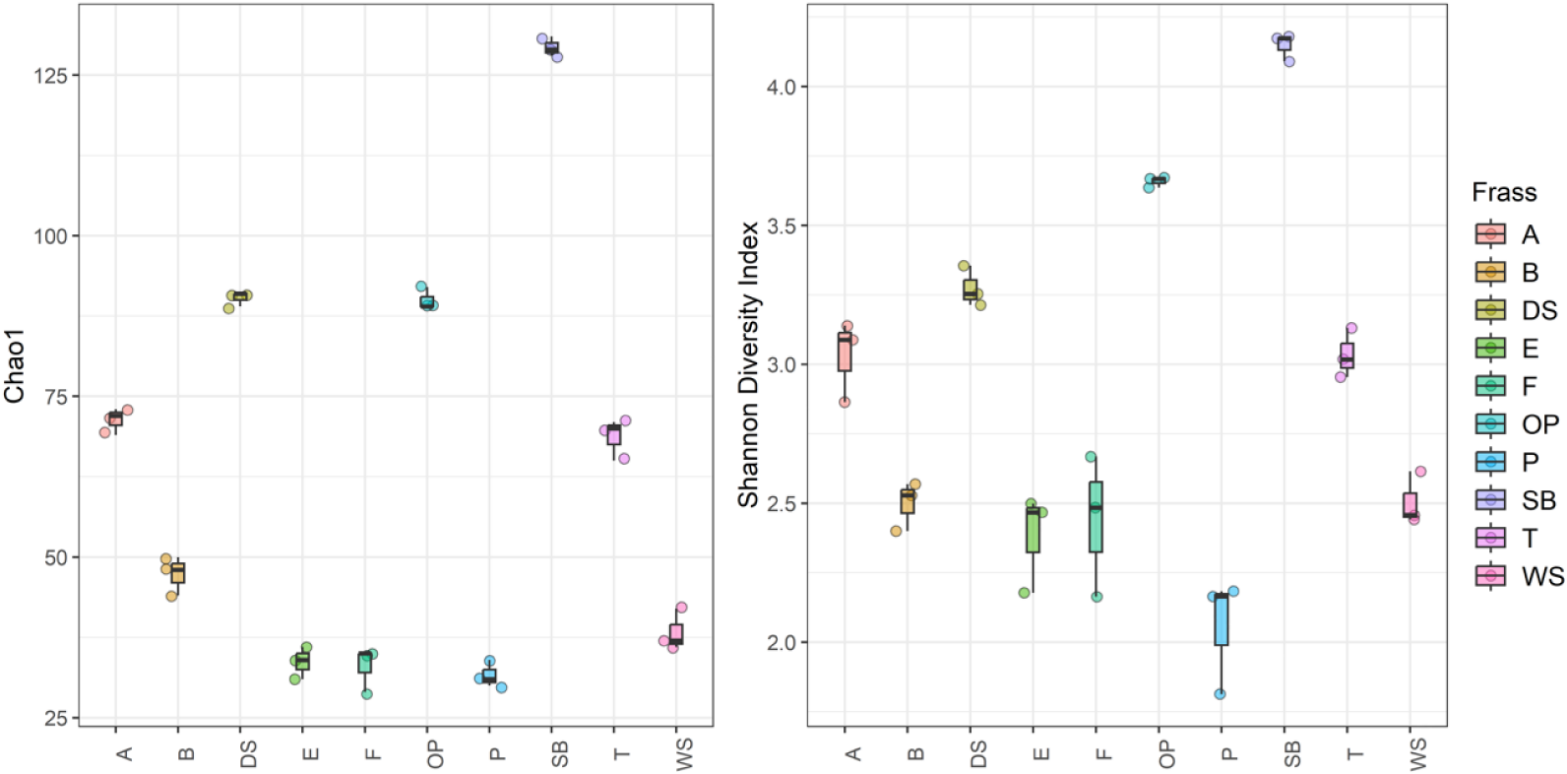
Alpha diversity matrices Chao1 and Shannon Diversity Index for 10 different frass types. Colors indicate the different frass types.

**Figure 11:**
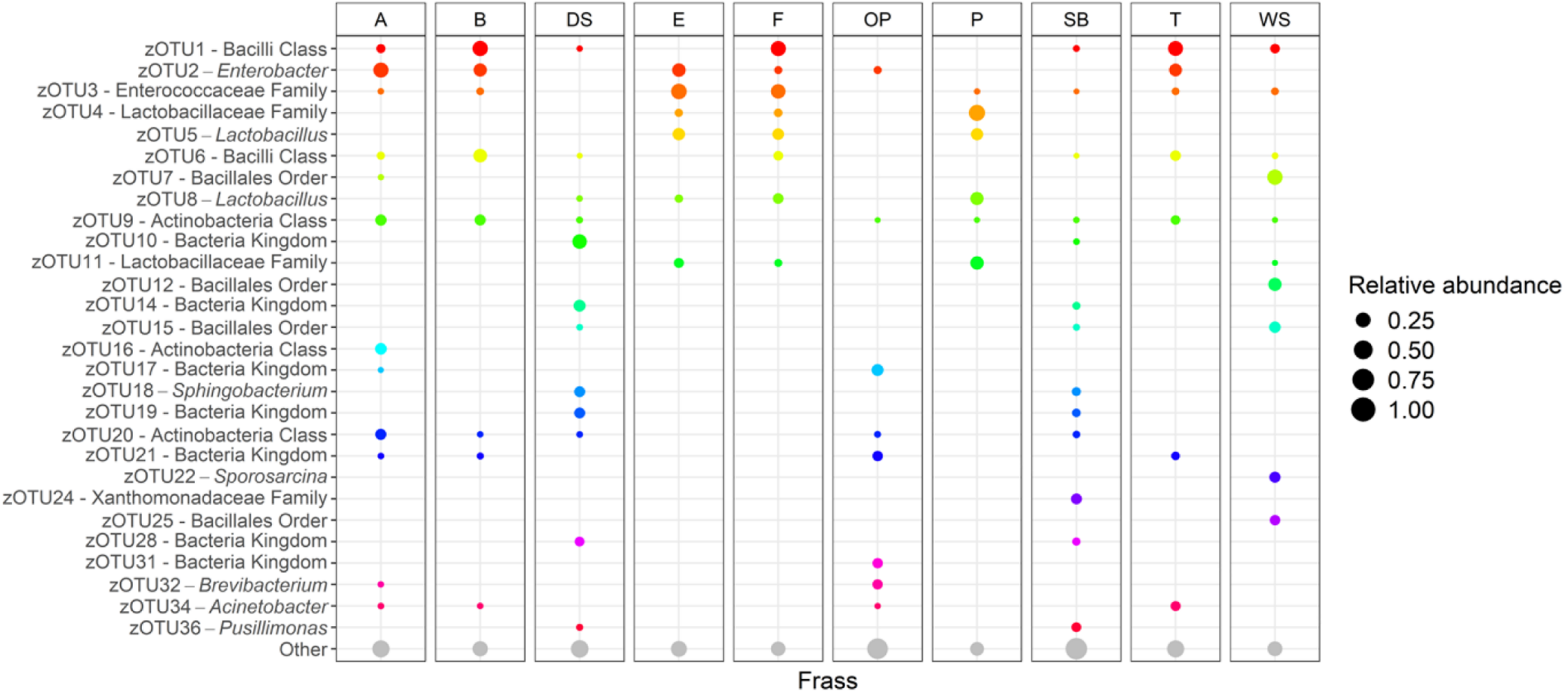
Mean relative abundance of zOTUs across frass type. The size of the dots represents the relative abundance of each of the three frass sample replicates for every frass type. Taxa were identified at the genus level or, when not possible, at the highest resolved taxonomic level. Only taxa with a relative abundance >5% in at least one frass type are shown (*n* = 28). All remaining zOTUs with <5% relative abundance were grouped under “Other”.

**Table 3:** Mean Relative Abundance by Frass Type^a^

Bacteria	A	B	DS	E	F	OP	P	SB	T	WS
zOTU1—Bacilli Class	4.0%	26.6%	0.1%	0.0%	25.9%	0.0%	0.0%	0.5%	24.8%	4.6%
zOTU2—*Enterobacter*	25.6%	16.3%	0.0%	17.6%	1.7%	1.6%	0.0%	0.0%	15.0%	0.0%
zOTU3—Enterococcaceae Family	0.3%	1.4%	0.0%	28.9%	23.1%	0.0%	0.1%	0.0%	1.3%	1.4%
zOTU4—Lactobacillaceae Family	0.0%	0.0%	0.0%	1.9%	2.7%	0.0%	32.6%	0.0%	0.0%	0.0%
zOTU5—*Lactobacillus*	0.0%	0.0%	0.0%	13.2%	10.0%	0.0%	12.8%	0.0%	0.0%	0.0%
zOTU6—Bacilli Class	1.8%	18.8%	0.0%	0.0%	4.7%	0.0%	0.0%	0.0%	7.8%	0.4%
zOTU7—Bacillales Order	0.1%	0.0%	0.0%	0.0%	0.0%	0.0%	0.0%	0.0%	0.0%	27.4%
zOTU8—*Lactobacillus*	0.0%	0.0%	0.2%	2.2%	7.8%	0.0%	17.1%	0.0%	0.0%	0.0%
zOTU9—Actinobacteria Class	9.1%	9.0%	0.5%	0.0%	0.0%	0.0%	0.0%	0.2%	4.1%	0.0%
zOTU10—Bacteria Kingdom	0.0%	0.0%	22.7%	0.0%	0.0%	0.0%	0.0%	0.3%	0.0%	0.0%
zOTU11—Lactobacillaceae Family	0.0%	0.0%	0.0%	5.7%	1.6%	0.0%	18.1%	0.0%	0.0%	0.0%
zOTU12—Bacillales Order	0.0%	0.0%	0.0%	0.0%	0.0%	0.0%	0.0%	0.0%	0.0%	16.4%
zOTU14—Bacteria Kingdom	0.0%	0.0%	11.5%	0.0%	0.0%	0.0%	0.0%	1.5%	0.0%	0.0%
zOTU15—Bacillales Order	0.0%	0.0%	0.4%	0.0%	0.0%	0.0%	0.0%	0.7%	0.0%	9.8%
zOTU16—Actinobacteria Class	10.8%	0.0%	0.0%	0.0%	0.0%	0.0%	0.0%	0.0%	0.0%	0.0%
zOTU17—Bacteria Kingdom	0.1%	0.0%	0.0%	0.0%	0.0%	11.5%	0.0%	0.0%	0.0%	0.0%
zOTU18—Sphingobacterium	0.0%	0.0%	7.8%	0.0%	0.0%	0.0%	0.0%	3.4%	0.0%	0.0%
zOTU19—Bacteria Kingdom	0.0%	0.0%	8.4%	0.0%	0.0%	0.0%	0.0%	2.6%	0.0%	0.0%
zOTU20—Actinobacteria Class	8.8%	0.2%	0.4%	0.0%	0.0%	0.5%	0.0%	1.0%	0.0%	0.0%
zOTU21—Bacteria Kingdom	0.3%	0.7%	0.0%	0.0%	0.0%	6.9%	0.0%	0.0%	2.5%	0.0%
zOTU22—*Sporosarcina*	0.0%	0.0%	0.0%	0.0%	0.0%	0.0%	0.0%	0.0%	0.0%	8.6%
zOTU24—Xanthomonadaceae Family	0.0%	0.0%	0.0%	0.0%	0.0%	0.0%	0.0%	8.3%	0.0%	0.0%
zOTU25—Bacillales Order	0.0%	0.0%	0.0%	0.0%	0.0%	0.0%	0.0%	0.0%	0.0%	6.9%
zOTU28—Bacteria Kingdom	0.0%	0.0%	5.3%	0.0%	0.0%	0.0%	0.0%	2.0%	0.0%	0.0%
zOTU31—Bacteria Kingdom	0.0%	0.0%	0.0%	0.0%	0.0%	7.0%	0.0%	0.0%	0.0%	0.0%
zOTU32— *Brevibacterium*	0.2%	0.0%	0.0%	0.0%	0.0%	6.3%	0.0%	0.0%	0.0%	0.0%
zOTU34—*Acinetobacter*	0.4%	0.2%	0.0%	0.0%	0.0%	0.2%	0.0%	0.0%	5.9%	0.0%
zOTU36— *Pusillimonas*	0.0%	0.0%	0.6%	0.0%	0.0%	0.0%	0.0%	5.4%	0.0%	0.0%
Other (<5%)	38.5%	26.6%	42.0%	30.5%	22.6%	66.0%	19.4%	74.1%	38.6%	24.4%

a For each frass type (columns 2 to 11), the relative abundance of three replicate samples (*n* = 3) was averaged. Taxa were identified at the genus level or, when not possible, at the highest resolved taxonomic level. Only taxa with a relative abundance >5% in at least one frass type are shown (*n* = 28). All remaining zOTUs with <5% relative abundance were grouped under “Other.”

Next, the short reads zOTU were identified using an NCBI nucleotide BLAST analysis, using the generated full 16S rRNA gene sequences of the six high performing plant-promoting bacteria as references (Section 3.2). For five zOTUs, the short reads of the V4 had a 100% match with one of the reference bacteria, and it can therefore be assumed that these zOTUs represent the six selected bacterial strains of interest in the frass samples (Supplementary Table 2). zOTU673 had the highest match with the *Peribacillus* sp. strain (98.4%) match; however, this zOTU673 contained nearly no reads and was removed during the filtering step. The relative abundance of the five remaining zOTUs for each of the three repetitions in the 10 different types of frass is shown in Figure 12.

**Figure 12:**
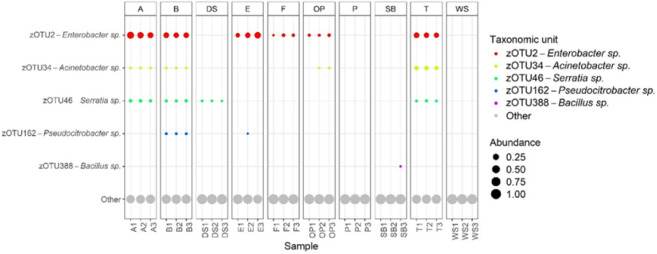
Relative abundance of zOTUs corresponding to PGP bacterial isolates. The size of the dots represents the relative abundance of each of the three frass sample replicates for each different frass type.

Interestingly, *Enterobacter* sp. (zOTU2) was found in most frass types (A, B, E, F, OP, and T), although the relative abundance was different in each group. Four of the high-performance bacteria (*Enterobacter* sp., *Acinetobacter* sp. (zOTU34), *Serratia* sp. (zOTU46), and *Pseudocitrobacter* sp. (zOTU162)) were found in all B frass samples. Frass types P and WS did not contain any of the bacteria of interest, and only one type of beneficial bacteria was found in the DS, SB, and F frass.

## 4 Discussion

Frass is not a homogeneous product; its composition and quality are strongly influenced by the feed substrates utilized throughout the rearing,^4^ as well as by postprocessing treatments applied for hygienic purposes,^46^ which can significantly alter its characteristics.^47^ Since BSFL can consume a variety of feeding substrates, the composition of frass can also vary. In a previous review paper,^2^ a comparison among the macro- and micronutrient compositions of different frass types was performed, showing how these differences can depend on the feeding substrate. This work investigates the microbial composition of different types of frass with a specific focus on the presence of bacteria exhibiting PGP characteristics. Plant–rhizobacteria interactions can enhance both the growth and protection of economically important crops. The presence of PGPM in frass, therefore, offers a potential agronomic and commercial advantage.

### 4.1 Screening Revealed Numerous Isolates with At Least One Plant Growth-Promoting Activity

A total of 149 bacterial isolates were collected and subjected to a preliminary screening phase to identify PGP activities. As illustrated in Figure 1, bacteria isolated from different types of frass display dissimilar characteristics; this confirms the variability between frass from different diets, which has also been reported by Wynants et al.^29^ and Osimani et al.^32^ Bacterial isolates recovered from HT frass exhibited variability across the evaluated parameters, in some cases showing either reduced or enhanced activity. For instance, in the case of bacteria isolated from frass type A, notable differences were observed: isolates from NT frass demonstrated a higher performance in ammonia and gibberellin production, whereas those from HT frass showed a greater phosphate solubilization capacity. Similar trends in functional variability were observed across all frass types, as illustrated in Figure 1.

To narrow down the list of bacterial isolates, the top 73 strains were selected based on their overall performance across the evaluated PGP traits. Strains exhibiting the highest activity in one or more of these assays were retained for subsequent molecular identification using 16S rRNA gene sequencing to reveal the different bacterial genera with PGP activities associated with the various types of frass. As stated in the results, the genera most commonly found in NT frass were also present in HT frass, indicating that heat treatment did not reduce the presence of bacteria with beneficial traits for plant growth. While the survival of *Bacillus* can be attributed to its ability to form heat-resistant spores, the persistence of the other nonspore-forming genera is particularly remarkable and suggests a notable tolerance to thermal stress.

This is further confirmed by dedicated testing of the ability of isolates to survive a thermal treatment of 1 h at 70 °C. This suggests there is a potential for their inclusion in integrated biofertilizer formulations where frass acts as both a microbial inoculum and an organic matrix. This finding is particularly relevant from an application standpoint, as it suggests that increasing their initial concentration prior to heat treatment may be a viable strategy to enhance the microbial load of the final product without compromising bacterial viability. This approach could help ensure both the sanitary safety and biological effectiveness of frass-based biofertilizers, though more research is needed on the effect of processing, on one hand, and the potential of these isolates to boost the growth of other plant species, on the other hand.

### 4.2 Most Identified Genera with *In Vitro* PGP Activity Frequently Associated with BSF Frass

Overall, the literature on the BSFL microbiota has revealed clear correlations between the gut and frass microbial community, where microbes related to the larvae become more dominant in the frass compared to the initial diet.^48^ Our study aligns with such results; the bacterial genera *Serratia*, *Peribacillus*, *Acinetobacter*, *Pseudocitrobacter*, *Bacillus*, and *Enterobacter*, which we isolated from frass, have already been reported in previous analyses of the microbiota associated with *H. illucens*. Regarding spore-forming bacteria, several studies have consistently reported *Bacillus* spp. as one of the most predominant in the gut microbiota of *H. illucens*, underscoring its central role in the digestive ecology of the species.^49–52^ In particular, Callegari et al.^50^ reported a notably high abundance of *Bacillus* spp. in the larval gut, suggesting that members of this genus are actively involved in the degradation of polysaccharides such as cellulose and starch. The genus *Enterobacter* has been frequently identified in the larval gut of *H. illucens* across several studies, including those by Callegari et al.,^50^ Gorrens et al.,^53^ and Cifuentes et al.^52^ Although less dominant, *Serratia* species were also detected in the gut of larvae, which suggests their stable presence, albeit in low amounts, in the microbial community.^52^ *Acinetobacter* is another genus commonly reported in the gut of larvae.^50^ The detection of these bacterial genera in the larval gut of *H. illucens*, as documented in various studies, along with their presence in the larval frass analyzed in this study, also supports a transfer of bacteria from the gut to the frass during the digestive process. In contrast, a literature review revealed no studies reporting the presence of the genera *Peribacillus* and *Pseudocitrobacter* in the gut microbiota of *H. illucens*. Therefore, the occurrence of these genera in the frass could be attributed to their presence in the initial feeding substrate.

### 4.3 Six Potent Isolates from Genera with Known PGP Activity Confirm the Suitability of the Screen

*Serratia* sp., *Peribacillus* sp., *Acinetobacter* sp., *Pseudocitrobacter* sp., *Bacillus* sp., and *Enterobacter* sp. were the six studied bacteria that showed the best traits and were explored in more detail. Their potential is also confirmed to a large extent by the literature. Numerous bacteria in the *Serratia* genus have been reported to increase plant growth by generating phytohormones, increasing nutrient availability, and offering defense against biotic and abiotic stress.^54^ *Peribacillus* has already been reported among PGPM showing high potential under stress conditions, particularly due to features such as nutrient solubilization and phytohormone production.^55^ Members of the *Acinetobacter* genus exhibit PGP characteristics, particularly phosphate solubilization, which contributes to improved plant performance under metal stress.^56^ Similarly, *Pseudocitrobacter* has been shown to promote plant growth by reducing heavy metal accumulation and enhancing phytohormone production and antioxidant activity in plants.^57^ Numerous *Bacillus* species have been shown to be beneficial to plants by increasing biomass, growth, and yield. They also aid in the synthesis of phytohormones, nitrogen fixation, phosphate solubilization, and enhanced nutrient availability.^58^ The genus *Enterobacter* has also been reported to exhibit excellent PGP characteristics.^59^ *Peribacillus polymyxa* was used as a control, but it did not solubilize phosphate or produce gibberellins, as reported by Weselowski et al.^45^

### 4.4 Differential Effects of Isolate Supplementation on Root Length and Root Hair Formation in *Arabidopsis* Seedlings

The effect of these isolates on plant growth was then observed in the *Arabidopsis* seedlings. When root length (Figure 6b) was compared with the number of root hairs (Figure 7) across the different treatments in these trials, a noteworthy observation was the negative correlation between these two parameters. Treatments that exhibited a greater root length generally showed a lower number of root hairs. For example, treatments with *Acinetobacter* sp., *Pseudocitrobacter* sp., *Enterobacter* sp., and *Paenibacillus polymyxa* showed shorter root lengths compared with the other treatments and the control. However, these same treatments led to a higher production of root hairs. Conversely, other treatments, such as those with *Serratia* sp., *Peribacillus* sp., and *Bacillus* sp., exhibited the opposite trend. Root formation of *A. thaliana* has been extensively studied, and the different steps of root hair development have been described.^60–62^ Hairs originate from selected epidermal cells influenced by genetic factors and local conditions affecting the loosening of cell walls to promote hair initiation and outgrowth.^62,63^ These processes can be influenced by the involvement of hormones (e.g., auxin, cytokinin, ethylene), local variations in nutrient or toxic elements, redox conditions, or pH.^64,65^ The results of our study highlight the potential of specific bacterial isolates associated with *H. illucens* frass to promote root hair formation. Notably, strains such as *Serratia* sp., *Peribacillus* sp., and *Bacillus* sp. not only demonstrated PGP traits—including phosphate solubilization and gibberellin production but also led to a significant increase in both root length and the density of root hairs per unit root length in controlled assays. These findings are consistent with literature indicating that bacterial production of phytohormones and solubilization of nutrients can lead to a modulated root system architecture.^66,67^ Interestingly, isolates such as *Acinetobacter* sp., *Pseudocitrobacter* sp., *Enterobacter*sp., and *Paenibacillus polymyxa* while also exhibiting certain PGP features, were associated with shorter root lengths but a higher density of root hairs per millimeter of root. This inverse relationship suggests that specific microbial metabolites may preferentially stimulate lateral root structures over elongation. For instance, gibberellin production has been linked to the modulation of cell elongation and differentiation in roots, but its combined effect with other substances (e.g., IAA, ammonia) could result in diverse morphological outcomes depending on concentration and interaction.^68^ Regarding root hair formation, while the role of IAA-producing bacteria in enhancing root hair density has been documented,^69^ less is known about the synergistic role of ammonia and gibberellins in this process. In our assay, *Acinetobacter* sp. and *Pseudocitrobacter* sp. both capable of gibberellin production induced the highest number of root hairs, even though their overall root length was limited. This observation may point to a mechanism in which energy is diverted toward lateral differentiation rather than elongation. From the perspective of plant–microbe interactions, this could be particularly advantageous: root elongation combined with an increased number of root hairs enhances the absorptive surface area, thereby improving the efficiency of water and nutrient uptake. Therefore, bacterial strains that promote the formation of a higher density of root hairs, even when associated with a slight decrease in overall root elongation, may represent desirable candidates for the development of bioinoculants under resource-limited conditions.

### 4.5 Substrate Dependence of Six Potent Isolate Abundance Explains Observed Variability in Frass Treatment Effects

Identification of the bacterial communities through 16S rRNA gene amplicon sequencing revealed clear differences among frass types. These variations likely reflect the ability of specific microbes to utilize specific nutrients in the diet, producing metabolites that enable them to thrive and dominate the microbiome. The diet dependency is evident from the absence of any single bacterium with both a high abundance and a wide prevalence across all samples. For example, the most abundant bacterium, the unidentified *Bacilli* zOTU1, accounts for approximately 25% of reads in frass types B, F and T, yet occurs at much lower levels in four other frass types (A, DS, SB, and WS), while being completely absent in three others (E, OP, and P). In most frass types, 60–80% of the bacterial community consists of fewer than 30 dominant microorganisms. A microorganism is defined as dominant if it is present in >5% in that specific frass type. In contrast, approximately 75% of the relative abundance in SB frass consists of species outside the 28 dominant taxa, highlighting the substantial variability in microbiome composition among frass types. These differences in the relative abundance were also evident among the six selected bacterial isolates across frass types. Notably, *Enterobacter* was detected in several frass types, specifically, A, B, E, F, OP, and T, and consistently exhibited the highest relative abundance among the targeted genera. *Acinetobacter* and *Serratia* were also detected in multiple frass types, including A, B, and T, with *Serratia* additionally present in DS and *Acinetobacter* also found in OP. However, the abundance of these two genera was clearly lower than that of *Enterobacter*. *Pseudocitrobacter* was exclusively detected in frass B and F, while *Bacillus* was found only in SB. Notably, frass B contained four different PGP genera: *Enterobacter*, *Acinetobacter*, *Serratia*, and *Pseudocitrobacter*, which indicates it might have the most diverse composition in terms of beneficial microbes. Overall, frass A, B, and T originating from larvae fed with artichokes, broccoli, and turnip greens, respectively, showed the highest potential in terms of content of the six characterized PGPM. In addition, these frass types were also associated with high loads overall of bacteria capable of growth on RMA (>6 log CFU/g). The increased presence of (potential) PGPM in frass derived from artichokes, broccoli, and turnip greens may be attributed, at least in part, to the biochemical composition of these substrates. Artichoke is characterized by high levels of soluble fiber (notably inulin),^70^ whereas Brassicaceae vegetables (e.g., broccoli and turnip greens) accumulate significant amounts of sulfurrich glucosinolates.^71^ These biochemical traits make these substrates chemically distinctive compared with other vegetable byproducts tested and could selectively favor microbial taxa adapted to metabolize or tolerate such compounds.The observed differences underscore the influence of larval diet on the microbial profile of the resulting frass. By strategically selecting feeding substrates for *H. illucens*, it may be possible to enhance the abundance of specific PGPM in the frass. Such an approach could be leveraged to develop functionally enriched frass-based biofertilizers tailored to agronomic needs, provided that the economic costs of substrate optimization remain justifiable. This study is among the first to provide direct evidence of microbes exhibiting PGP traits in frass derived from BSFL reared on various dietary substrates. Therefore, this study provides evidence supporting the hypothesis that PGP microbes present in frass may partially explain the beneficial effects on plant growth and health reported in previous literature. Our findings also highlight that the composition of the larval diet significantly influences the diversity and abundance of PGP microbes present in BSF frass. This could explain the large variability between studies when testing the effect of frass supplementation on crops. Interestingly, most of the PGPM isolated in this study demonstrated an unexpected ability to survive applied heat treatment. This suggests that frass could retain beneficial microbial activity even after mandatory heat treatments. Further studies are needed to deeply understand the molecular mechanisms involved and to determine whether coapplication of such strains could lead to additive or synergistic benefits in plant nutrient uptake, particularly under water- or nutrient-limited conditions.

## Supplementary Material



## Data Availability

The data sets used and/or analyzed during the current study are available from the corresponding author on reasonable request.

## Notes

Includes supplementary figures and tables providing additional data in support of the analyses discussed in the main text; schematic overview of the experimental workflow, bacterial counts, and plate images; taxonomic comparison based on 16S rRNA gene analysis; and plant growth-promotion assays (PDF)

## Glossary

BSF: Black Soldier Fly
PGPM: Plant Growth-Promoting Microorganisms
IAA: Indole-3-Acetic Acid
BSFL: Black Soldier Fly Larvae
6-DOL: Six-day-old Larvae
DS: Standard Diet
HT: Heat-Treated
NT: Nontreated
DM: Dry Matter
RMA: Rhizosphere-Mimicking Agar
PGP: Plant Growth-Promoting
LB: Luria–Bertani
PCA: Plate Count Agar
zOTUs: Zero-Radius Operational Taxonomic Units
RH: Relative Humidity
CFU: Colony-Forming Unity
