# Supplementary material for: Selection and In Vitro Assessment of Plant Growth-Promoting Bacteria from Black Soldier Fly (Hermetia illucens) Frass
Source: ACS Agric Sci Technol. 2026 Feb 12;6(3):489–504. doi: 10.1021/acsagscitech.5c00811 (PMC12997410; doi:10.1021/acsagscitech.5c00811)

Supporting Information

Selection and *In Vitro* Assessment of Plant Growth-Promoting Bacteria from Black Soldier Fly (*Hermetia illucens*) Frass

Giovanni Lomonaco[†], Jeroen De Smet[*‡], Freek IJdema[‡], Johan Ceusters[§], Francesco Iannielli[†], Rosanna Salvia[†‖], Mariana Amato[⊥], Carmen Scieuzo[†‖], Patrizia Falabella[*†‖]

[†] Department of Basic and Applied Sciences, University of Basilicata, Potenza, 85100, Italy
[‡] Research group for Insect Production and Processing, Department of Microbial and Molecular Systems, KU Leuven, Geel, 2440, Belgium
[§] Department of Biosystems, Research Group for Sustainable Crop Production and Protection (SusCroPP), KU Leuven, Geel, 2440, Belgium
[‖]Spinoff XFlies s.r.l, University of Basilicata, Potenza, 85100, Italy
[⊥] Department of Agriculture, Forestry, Food and Environmental Sciences, University of Basilicata, Potenza, 85100, Italy

*Email: jeroen.desmet@kuleuven.be.

*Email: patrizia.falabella@unibas.it.

This document contains: 5 pages, 4 Figures and 2 Tables.

Supplementary Table 1. Composition of Rhizosphere Mimicking Agar (Brescia et al., 2020).

**Type**	**Ingredients**	**Concentration (g/l)**
**Synthetic root exudates**	Citric acid	0.010
	Fructoseb	0.039
	Glucose	0.039
	Glutamic acid	0.019
	L-Alanine	0.019
	L-Serine	0.023
	Lactic acid	0.001
	Succinic acid	0.010
	Sucrose	0.037
**Recalcitrant organic carbon sources**	Cellulose	0.079
	Humic acids	0.033
	Lignin	0.065
	Starch	0.017
**Salts**	CuSO4 5H2O	0.005
	KCl	0.499
	KH2PO4	0.680
	Fe2(SO4)3	0.031
	MgSO4 7H2O	0.493
	MnSO4	0.001
	Na2MoO4 2H2O	0.008
	(NH4)2SO4	0.002
**Cycloheximide**		0.1

Supplementary Table 2. Taxonomic units found in the 16S rRNA gene amplicon analysis of the frass samples were compared with the full 16S sequences of the six bacterial isolates of interest. Names of the bacterial isolates and the best matching sequences from the amplicon sequencing analysis of the frass are shown in column 1 and 2, respectively. Column 3 shows the percentage that the short reads matched with the bacteria isolate full 16S rRNA gene sequences.

Bacteria	Short read ID frass	Percentage identification match (%)
Enterobacter sp.	zOTU2	100.000
Acinetobacter sp.	zOTU34	100.000
Serratia sp.	zOTU46	100.000
Pseudocitrobacter sp.	zOTU162	100.000
Bacillus sp.	zOTU388	100.000
Peribacillus sp.	zOTU673	98.400

Supplementary Figure 1. A schematic and simplified overview of the experimental procedures, which are described in full detail in the Materials and Methods section. I: isolation of bacteria from frass; II: screening for evaluation of PGPM trait from bacteria selected from frass; III: in vivo test with *Arabidopsis thaliana*.

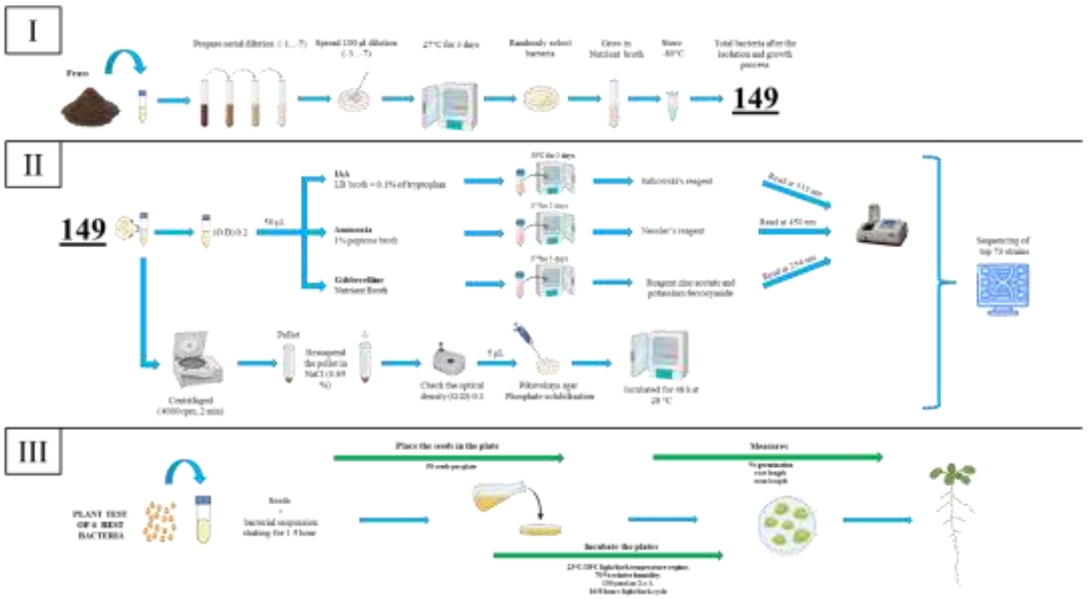

Supplementary Figure2. Total bacterial counts on RMA (expressed as log CFU/g dry matter (dm)) from ten different types of frass, either untreated (blue bars) or heat-treated (orange bars). Frass types include: DS = Gainesville diet, WS = whey + seeds, SB = spent barley, OP = olive pomace, P = peppers, E = eggplant, T = turnip greens, A = artichokes, B = broccoli, F = fennel. Each bar represents the count value of a single replicate. Heat treatment resulted in a general reduction of bacterial load, although variability among substrates was observed. The data shown in the graph are presented for descriptive purposes only for the bacterial count on RMA and were not subjected to statistical testing. Heat treatment at 70 °C generally reduced the presence of bacteria grown on RMA.

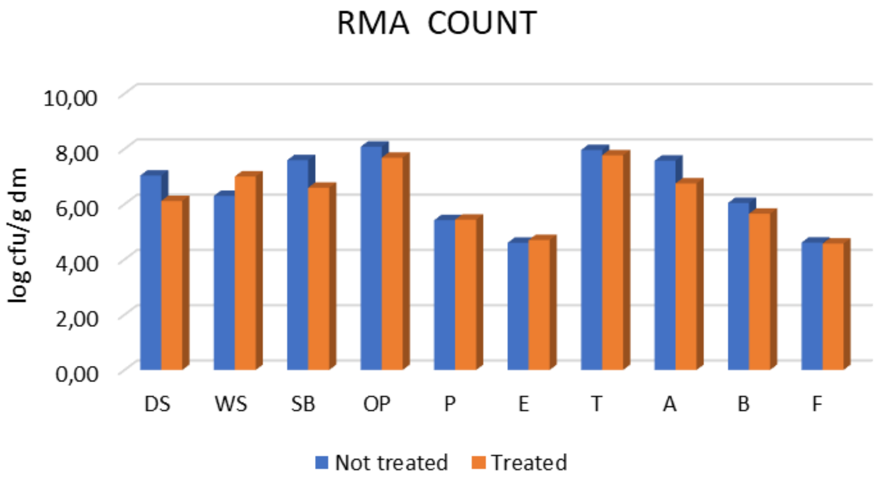

Supplementary Figure 3. The figure illustrates the RMA plates displaying the growth of bacterial colonies. Different letters represent different dilution of frass extract (A: 10-4; B: 10-5)

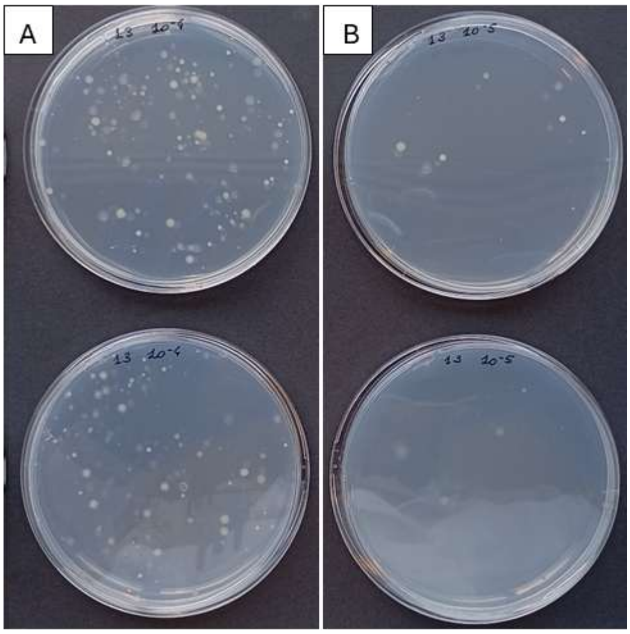

Supplementary Figure 4. Germination percentage of *Arabidopsis thaliana* seeds treated with different plant growth-promoting bacteria (PGPB), compared to the control (CTRL). Data are presented as mean of 3 replications with 50 seeds per plate.

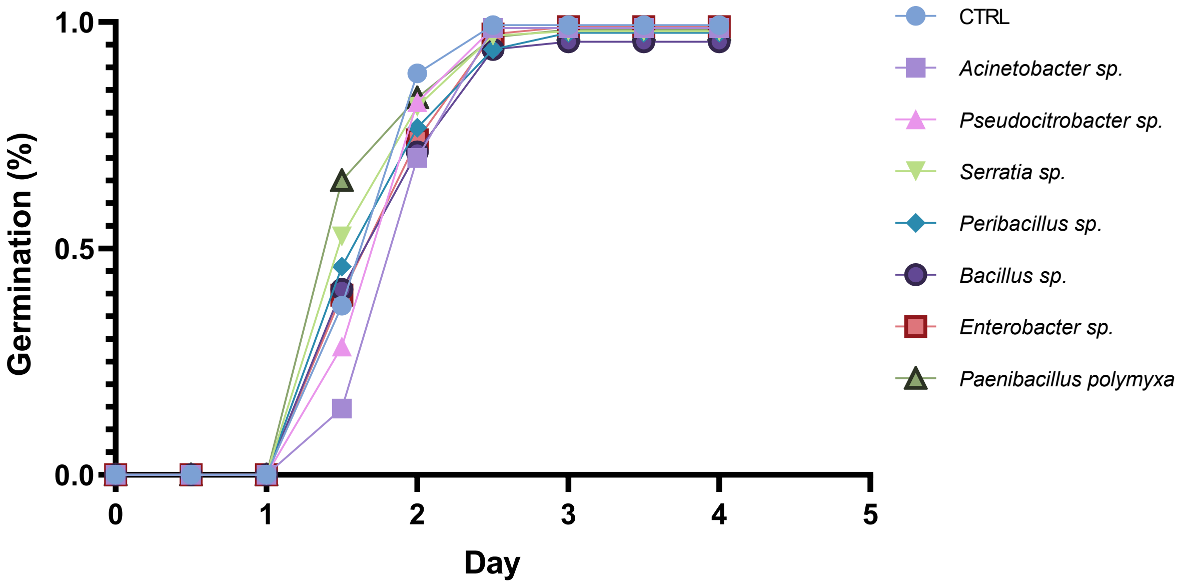

Supplement: Supplementary file 1 [file as5c00811_si_001.pdf]
